# Percentile Distribution of Habitual-Correction Visual Acuity in a Sample of 1500 Children Aged 5 to 15 Years in Italy

**DOI:** 10.3390/pediatric17040085

**Published:** 2025-08-11

**Authors:** Alessio Facchin, Marilena Mazzilli, Silvio Maffioletti

**Affiliations:** 1 Neuroscience Research Center, Department of Medical and Surgical Sciences, Magna Graecia University, 88100 Catanzaro, Italy; 2 Institute of Research and Studies in Optics and Optometry, 50059 Vinci, Italy; silvio.maffioletti@gmail.com; 3 Degree Course in Optics and Optometry, University of Torino, 10124 Torino, Italy; marilena.mazzilli84@gmail.com

**Keywords:** visual acuity, children’s vision, vision screening, LEA symbol, percentile, normative data

## Abstract

**Background:** Early identification of visual disorders in children is essential to prevent long-term visual impairment and support academic development. Despite the recognized importance of visual screenings, no universal consensus exists on which visual parameters or threshold values should be used, particularly for measuring visual acuity (VA) in pediatric populations. **Objectives:** This study aimed to develop age-related percentile norms for VA using LEA symbol charts. **Methods**: A sample of Italian schoolchildren aged 5 to 15 years (*n* = 1510) participated in the study. Data were collected retrospectively from school-based vision screenings conducted across 12 schools in the Lombardy and Piedmont regions from 2010 to 2019. Monocular and binocular VA were measured at 3 m using a standardized LEA symbol chart, and values were scored letter-by-letter on a LogMAR scale. Smoothed percentile curves were derived using Box–Cox, Cole, and Green distribution modeling and regression analysis. **Results:** The results showed a non-linear improvement in VA with age. Compared to prior studies, LEA symbols yielded slightly lower VA scores, reinforcing the need for chart-specific norms. The 50th percentile VA improved from approximately +0.07 LogMAR at age 6 to about −0.09 LogMAR at age 15. **Conclusions**: These findings highlight the importance of age-specific, chart-specific, and statistically robust reference data for VA screening in children. The derived percentile tables offer a more sensitive tool than fixed cut-offs for identifying visual anomalies and tailoring clinical interventions. This work contributes to standardizing pediatric VA screening practices and improving early detection of visual deficits.

## 1. Introduction

Early diagnosis of visual disorders is essential to prevent permanent vision problems, guide treatment decisions, and support a child’s academic development. Undetected visual impairments can disrupt the normal development of visual perception and significantly affect its quality [1]. This risk is particularly critical in conditions such as amblyopia, which emerge in early childhood and, if left untreated, can lead to irreversible deficits in visual function [2,3]. Visual screening programs play a vital role in the early identification of such disorders [4].

Despite their recognized value, there is no universal scientific agreement on which visual parameters should be assessed during screening [5,6]. Recent initiatives, such as those from the Amblyopia Treatment Study group, have contributed to the development of standardized testing protocols, highlighting the importance of consistency in pediatric vision assessment. To ensure that measurements are accurate, reliable, and reproducible, both clinical practice and research must adopt uniform evaluation principles [7].

The variability of screening criteria between different countries and healthcare centers highlights the need for a uniform evaluation protocol. It would therefore be advisable to identify universal criteria to use, such as the type of test, the correct distance, lighting conditions, and administration time. All of these factors may affect the results [8].

A wide consensus is needed on the most fundamental parameter to consider, which is the measurement of visual acuity (VA) [3,5,6,9]. In this context, the term “cut-off” refers to the threshold value used to determine whether a person’s visual acuity is considered normal or indicates a possible anomaly that requires further investigation or treatment. However, no consensus was raised on the cut-off for use in children. Since the development of the visual system differs with age and environment, VA normality criteria must be established based on age [10]. Currently, another problem is the lack of standardization of visual acuity measurement, with only two main standards being approved, normative [11] and scientific [12], but they are not targeted specifically for children. Coherently, a single VA value for children’s visual screening is not valid at all ages [13,14]. Considering visual acuity in children, the results vary depending on the child’s age, the chart used, and the methodology employed to establish the threshold [7].

An essential aspect of visual acuity assessment in children is the choice of the correct optotype (LEA, HOTV, Landolt C, Tumbling E). LEA optotypes are a standardized and reliable solution for measuring visual acuity in children who cannot read and are frequently used at pediatric ages [15]. They consist of four symbols (circle, square, apple, and house) built in different sizes, but with the same confusability. A study has highlighted differences in VA thresholds in the use of different optotypes, procedures, and presentation modalities, such as single symbol, line, block, and chart presentation. Specifically, compared to SLOAN letters, LEA symbols show lower VA by about one line [16].

About VA thresholds in children, various studies have proposed different cut-offs. Becker et al. [17], using Lea symbols, showed a higher applicability in children compared to Landolt C and set a threshold value of +0.10 LogMar. Other authors defined <+0.30 [3] and <+0.20 LogMAR [4]. In defining normative monocular visual acuity for early treatment of diabetic retinopathy study charts in emmetropic children 5 to 12 years of age, the 95% lower prediction limits were +0.38 LogMAR for 5-year-olds and +0.30 LogMAR (20/40) for 6- to 12-year-olds [18]. However, these cut-offs cannot be compared directly due to the aforementioned multiple factors that influence the threshold of VA.

There are several advantages to using percentile scoring over a fixed cutoff, including the ability to directly identify rare test outcomes, increased sensitivity to subtle changes in performance, and broader international applicability [19]. Percentiles are commonly used in developmental neuropsychology, healthcare, and education as they facilitate clear and standardized communication among professionals [20]. In the context of pediatric visual acuity (VA) assessment, percentile curves offer a more nuanced and sensitive alternative to rigid threshold criteria [21]. By capturing gradual changes over time, they support more individualized and age-appropriate clinical interventions. Percentile curves enable clinicians to compare a child’s VA against normative developmental data, aiding in the detection of values that fall significantly below the expected range and may warrant further diagnostic evaluation. For example, Navas-Navia [8] generated VA percentile curves for children aged 3 to 12 years, demonstrating an age-related improvement in visual acuity. However, these graphs were not based on a continuous curve function; instead, they consisted of discrete age-based steps, resulting in fluctuations from 3-month to 3-month steps and lacking a true smooth curve-fitting model.

The aim of this study is to construct the percentile age-related norms for VA using LEA symbol charts in a sample of 1510 children aged 5 to 16 years, aiming to provide reference data useful for clinical evaluation and vision screening. Through analysis, the goal is to outline the normal development of visual acuity in this age group, highlight variations related to growth, and increase the availability of normative references for LEA charts in school-age children using a robust methodology.

## 2. Materials and Methods

### 2.1. Participants

Data were collected retrospectively from a series of vision screenings previously performed in 12 different schools in the Lombardy and Piedmont regions of Italy from 2010 to 2019. These screenings were performed with the first aim of visual screening by about 30 trained examiners with the supervision of the authors. For this reason, not all data was available for all participants (binocular VA). Inclusion criteria were the minimum measurement of monocular visual acuity of both eyes.

Exclusion criteria were the presence of a diagnosed actual or past visual, neurological, or psychiatric disorder. Initially, a sample of 1550 children was collected, but the following were removed: 14 for lack of two monocular VA values, 5 for lacking one monocular VA value, 14 for lack of age values, 1 child who was 16 years old, and 6 for aberrant values outside of a scale of VA. The final sample was composed of a total of 1510 children. The final mean age was 8.8, the SD was 3.2, and the range was 5–15. Relatives of all children signed informed consent to participate in the visual screening and signed the option to participate in this research. This study was conducted in accordance with the principles of the Declaration of Helsinki. The first school (IC Muzio, Bergamo, Italy) provided ethical approval (auth. n. 23/2010; 5 April 2010). Each school thereafter ratified the ethical authorization, provided parental permission, and informed consent in accordance with national assessment guidelines.

### 2.2. Visual Acuity Measurements

The measurement of habitual distance visual acuity was performed by using a Lea Symbols^®^ 15 line chart with 100% spacing (Good-Lite 250160, https://store.good-lite.com/products/250160 (accessed on 1 August 2025)) at a distance of 3 m. This chart follows a logarithmic progression in steps of 0.1 LogMAR from +1.0 LogMAR to −0.3 LogMAR. As requested, the eye chart was positioned 3 m from the eyes of the child at the same height. The measurements were performed in a quiet, uniformly lit room with a luminance > 350 lux at the level of the eye chart.

The scoring procedure followed the letter-by-letter visual acuity measurement procedure [22,23]. Firstly, participants were requested to name vertically the fourth, second, and third columns of symbols, respectively, for OD, OS, and OU measurement from the largest to the smallest symbol (top to bottom). One experimenter indicated each symbol on the eye chart while the second experimenter stayed near the child and covered the other eye with an eye patch and maintained a distance when performing monocular evaluations. When an error was made, the standard horizontal symbol reading line by line, starting from the previous line in which the participant made an error, was performed. The entire 5 symbols on each line were read up by following these criteria to establish the VA value. The value corresponding to the smallest line in which at least three out of five targets were correctly named by the participant was recorded as the VA value. The number of symbols recognized and verbally reported (i.e., 3/5, 4/5, and 5/5) was then recorded, along with the number of letters read on the following lines (up to two). In the final adjusted VA value, all symbols read correctly (−0.02) and missed (+0.02) were considered, giving a final single value in each step of 0.02 LogMAR (e.g., +0.1 LogMAR 3/5 +1 was transformed to +0.12). All VA values were analyzed in the LogMAR scale.

### 2.3. Procedure

Testing was performed in a quite well-illuminated room at school, and children were tested in groups of two or three. One child performs the VA measurement while the other two perform the other screening measurements. Monocular (right and left eye) and binocular habitual visual acuity were measured in that order. Children who used glasses were instructed to wear them as usual. Prior to VA measurement, the LEA symbols were presented to the child using a specific near card in which symbols are printed very large (about 5 cm each) and described to familiarize. Prior to VA measurement, these symbols were named by children to assess their comprehension. VA was tested in a fixed order, first for OD, then OS, and OU, when required. Binocular visual acuity was assessed in approximately half of the participants, in compliance with the specific requirements established by the organization. The VA values were recorded on a specific score sheet. Refractive assessments were not performed because the aim was to evaluate habitual-correction visual acuity (HCVA) within a screening context.

### 2.4. Statistical Analyses

A series of descriptive analyses was performed to describe the sample acquired and the values of VA obtained. Age was expressed in years for descriptive reporting and percentile tables. Conversely, age expressed in months was used for regressions and percentile calculations. Comparison between the mean value of VA was performed by a paired t-test or Wilcoxon signed rank test, depending on the distribution of data. Normality was assessed by observing skewness and kurtosis. Normality was considered if Kurtosis was |1| and Skewness was |3| [24]. A series of regressions was performed to assess the best relationship between age as an independent variable and VA as a dependent variable. The best transformation of the dependent variable was assessed using bivariate regression and different transformations (logarithmic, quadratic, cubic, inverse, square root, and polynomial). Model selection was based on the lowest AIC value [25,26]. Calculations were performed separately for OD, OS, and OU. The mean difference between monocular and binocular VA was calculated. Smoothed percentile curves were then calculated by fitting a Box–Cox, Cole, and Green (BCCG) distribution using a generalized additive mixed model. Percentile curves were then derived from this model, and percentile tables were calculated. All analyses were performed using the R statistical environment 4.3.2 and specific packages [27].

## 3. Results

First, descriptive analyses of the sample were conducted. Table 1 presents the demographic characteristics of the sample, while Table 2 reports the descriptive results of visual acuity (VA).

The sample distribution across age showed that only some children were the age of 5, while the large part presented an age between 6 and 11 years old, with a minimum percentage of 15-year-old children. The detail of sex was reported in 1168 participants (77.4%), subdivided into 548 females (46.9%) and 620 males (53.1%). There is no association between age and sex (χ^2^ (10) = 13.33, *p* = n.s.).

As reported, Overall VA ranges between +0.92 and −0.30 LogMAR. The skewness and kurtosis of all three VA values exceed the references and, consequently, are not normally distributed. The comparison between OD and OS shows substantially similar values between OD and OS (V = 3.3 × 10^5^, *p* = 0.22). Unfortunately, binocular visual acuity was reported only in 41.8% of the sample. A Wilcoxon signed rank test comparing the binocular VA to the mean of the monocular one showed a significant increase in VA (V = 1.1 × 10^5^, *p* < 0.0001) with a mean value higher than the monocular of −0.05 LogMAR) equal to two and a half symbols.

In assessing the best model that considers VA and age in months, the best model with a lower AIC result is as follows:

VA_OD = −0.11
+ 13.35 × (1/Age_months)

VA_OS = −0.087
+ 11.037 × (1/Age_months)

VA_OU = 0.60 −
0.13 × log (Age_months)

**Figure 1:**
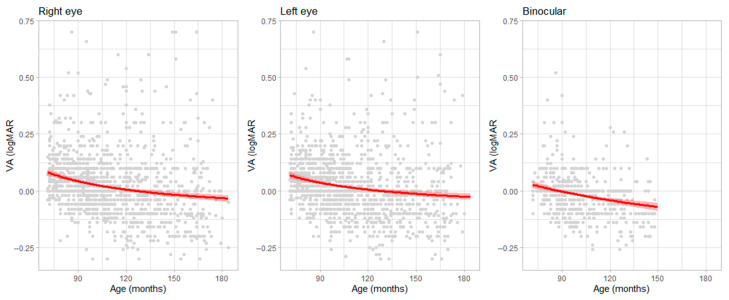
Best regression models that show the relationship between age (*x*-axis) and HCVA (*y*-axis). The red line represent the regression line, the shaded areas represent the 95% confidence interval of the regression and each grey point a single VA values. Datapoint could be overlapped.

Figure 1 displays the fitted models for the right eye (OD), left eye (OS), and both eyes (OU), illustrating a non-linear improvement in visual acuity (VA) across age.

**Figure 2:**
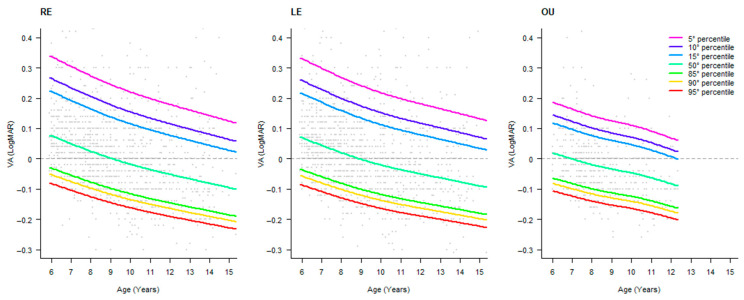
Percentile smoothed distribution of HCVA values across age for the right eye (OD), left eye (OS), and binocular (OU). Points represent the scatterplot of VA values across age, and lines represent specific percentile curves. The horizontal dotted line represents a value of zero LogMAR. On the *y*-axis, lower values represent better acuity.

Coherently, the fitting with a BCCG distribution showed a similar trend and an asymmetrical distribution of percentiles between the upper and the lower part of the distribution. The OD and OS distributions are quite similar, and, obviously, the binocular VA showed higher values of VA. Data are depicted in Figure 2. The advantages of using these models are to obtain smoothed results, giving far from small oscillations in VA values and non-uniform data in one area of the curve. Based on these models, the respective tables of percentiles were developed and reported in Table 3, Table 4 and Table 5.

## 4. Discussion

This study aimed to define percentile age-related norms for VA using a paper LEA symbol chart in a sample of more than 1500 Italian children aged 5–15. Overall, the results show a non-linear increase in VA in the considered age range, underlying the need for age-specific norms that were calculated and reported in specific tables.

In several studies, lower acuity values have been reported by LEA eye charts. Our chart is a simple LogMAR 100% spaced chart used commonly in clinical practice, which motivated us to provide normative data. As expected, the mean VA value changes with age, showing a better fit with an inverse formula for monocular VA and a natural logarithmic formula for the binocular one.

In light of these results, as well as abnormal skewness and kurtosis, a nonparametric model was needed to fit these data. In fact, the BCCG transformation has been shown to be the best model to fit these data [28].

Observing the tables, taking as a reference the 50th percentile, the values started from +0.076 LogMAR (OD) and +0.070 LogMAR (OS) at 6 years, becoming −0.096 LogMAR (OD) and −0.090 LogMAR (OS), giving an improvement over the years of 0.16 LogMAR, more than 1 and a half lines.

It is necessary to remark and report that these values have taken into account habitual-correction visual acuity. Consistently, this included the (small) improvement of VA and the better ability to resolve crowding from 6 to 15 years [23], but more importantly, they have given the awareness of uncorrected refractive errors to children and relatives. These data were collected during visual screenings with the primary purpose of showing these problems with the possibility of remediation. It becomes obvious that, in the case of refractive error, the problem is the first diagnosis [29].

While there are many reports of percentile curve improvement for children (e.g., [20]), few studies have taken into account vision [30], and only one took VA into account [8]. However, as previously remarked, the authors have not fitted the data with a robust statistical model and reported only data month by month.

Few studies have reported VA norms using LEA charts. Undoubtedly, the LEA charts give lower VA values [16]. Consistently, Becker et al. [17] have shown that, using a LEA Symbols Single Symbol Book in age groups > 36 months, the 10% percentile for VA of the “better” eyes was between +0.1 and 0.00 LogMAR. This result is incredibly higher than those found in this research of about 0.26 and is probably derived from the different charts used and the consideration of only the “better” eyes, which has distorted the measure by excluding low values.

In the study of Navas-Navia [8], the following results were observed in children between the ages of 3 and 11: at 6, they found a 5th percentile VA of +0.20 LogMAR and at the 50th percentile of 0.00 LogMAR, and at 11, they found a 5th percentile VA of +0.20 LogMAR and at the 50th percentile, −0.10 LogMAR. Our results showed that at 6, the 5th percentile of +0.33 LogMAR and at the 50th percentile, +0.07, and at 11, the 5th percentile was +0.20 LogMAR and at the 50th percentile, −0.04 LogMAR. Considering the low tail of the distribution, in the cut-off area, our results are lower for 6-year-olds and equal for 11-year-olds. Considering the median value, we found a lower VA of about one line. This is perfectly in line with the other literature that has found lower LEA symbols [16,31] using a similar HOTV-SLOAN chart.

Dobson [18], using ETDRS charts in 252 emmetropic children 5 to 12 years of age, found that for the best corrected (BCVA), a lower 95% prediction limit for determining whether a child has visual acuity within the normal range was +0.38 for 5-year-olds and +0.30 for 6- to 12-year-olds. The first value is perfectly in line with the present results, and the second is lower. Using the Sonksen logMAR test of visual acuity, the authors have determined the norms for children aged 2 to 9 years. They correctly used percentile-fitted curves for VA and set the 5th percentile to +0.18 LogMAR for children > 5 years. As a result, this value is lower than we found [32].

The percentile tables are useful in diagnosis when also taking into account that the International Classification of Functioning requires the percentile threshold of visual function abilities, including VA [33].

A major strength of this study lies in the development of HCVA percentile reference tables based on data collected from a large and diverse sample of over 1500 children. These values were obtained using a straightforward and accessible assessment method, an eye chart combined with a letter-by-letter scoring approach, making the findings both practical and easily applicable in clinical and educational settings.

The present study, which reports a large cohort of subjects, can greatly enhance the precision, relevance, and applicability of normative data concerning visual acuity, and, moreover, is reported in open access. It improves clinical practice and screening protocols. Evidence strongly supports moving away from historical standards (e.g., 10/10) towards data-driven benchmarks based on large-scale, representative samples.

Despite these strengths, the study also presents certain limitations. The dataset includes fewer binocular VA measurements compared to monocular ones, which may limit the generalizability of the results in contexts where binocular vision plays a critical role. Additionally, data on refractive status and best-corrected visual acuity (BCVA) were not collected, which could have provided a more comprehensive understanding of the children’s visual capabilities. However, the inclusion of refractive measurements was beyond the intended scope of this study, which focused specifically on uncorrected HCVA in a screening context.

Future research should aim to integrate refractive data and BCVA assessments to enrich clinical relevance and further validate their combined utility in identifying visual deficits. Furthermore, including younger children aged 3 to 5 years, using the same methodology and criteria, could enhance the generalizability and relevance of the findings. Indeed, the inclusion of participants spanning the entire lifespan—not only children but also adults—could potentially contribute to the development of updated normative data, offering a more comprehensive framework for evaluating visual function from early childhood through older adulthood.

## 5. Conclusions

The study provided monocular and binocular VA curves and percentile tables for children aged 6 to 15 years using a crowded LEA eye chart. With the proposed tables, visual impairments can be identified more accurately, and early intervention can be provided. The data can also be used to monitor visual development over time and compare results across different populations, methods, and procedures.

## Figures and Tables

**Table 1:** Age subdivision of the sample: distribution of participants across defined age groups, highlighting the number of individuals within each age range, percent, and cumulative percent.

Age (Years)	Frequency	Percent	Cumulative Percent
5	7	0.46	0.46
6	235	15.56	16.03
7	275	18.21	34.24
8	287	19.01	53.25
9	175	11.59	64.83
10	233	15.43	80.26
11	108	7.15	87.42
12	72	4.77	92.19
13	80	5.30	97.48
14	35	2.32	99.80
15	3	0.20	100.00
Missing	0	0.00	
Total	1510	100.0	

**Table 2:** Descriptive statistics of visual acuity (VA) in the sample: includes measures such as mean, standard deviation, minimum, and maximum values.

	OD	OS	OU
Valid	1510	1510	631
Missing	0	0	879
Mean	0.02	0.02	−0.02
SD	0.13	0.13	0.09
Minimum	−0.30	−0.30	−0.26
Maximum	0.74	0.92	0.52

**Table 3:** Visual acuity percentile distribution table across age for OD. Data is expressed in LogMAR.

Age (Years)	Percentile Rank						
	5	10	15	50	85	90	95
6	0.337	0.265	0.222	0.076	−0.031	−0.052	−0.082
7	0.305	0.234	0.192	0.050	−0.055	−0.075	−0.104
8	0.274	0.205	0.164	0.025	−0.077	−0.097	−0.125
9	0.245	0.178	0.138	0.002	−0.098	−0.118	−0.145
10	0.220	0.154	0.115	−0.018	−0.116	−0.135	−0.162
11	0.198	0.134	0.095	−0.036	−0.132	−0.151	−0.177
12	0.179	0.115	0.077	−0.052	−0.146	−0.165	−0.191
13	0.160	0.097	0.060	−0.067	−0.159	−0.178	−0.203
14	0.142	0.080	0.044	−0.081	−0.173	−0.191	−0.216
15	0.124	0.063	0.027	−0.096	−0.186	−0.204	−0.228

**Table 4:** Visual acuity percentile distribution table across age for OS. Data is expressed in LogMAR.

Age (Years)	Percentile Rank						
	5	10	15	50	85	90	95
6	0.330	0.258	0.215	0.070	−0.036	−0.058	−0.087
7	0.299	0.228	0.186	0.044	−0.059	−0.080	−0.108
8	0.268	0.200	0.159	0.020	−0.081	−0.101	−0.129
9	0.241	0.174	0.134	−0.002	−0.101	−0.121	−0.148
10	0.218	0.152	0.112	−0.021	−0.118	−0.137	−0.164
11	0.198	0.133	0.094	−0.037	−0.132	−0.151	−0.178
12	0.181	0.117	0.079	−0.050	−0.145	−0.163	−0.189
13	0.165	0.102	0.064	−0.063	−0.156	−0.175	−0.200
14	0.148	0.086	0.049	−0.077	−0.168	−0.186	−0.212
15	0.132	0.070	0.034	−0.090	−0.180	−0.198	−0.223

**Table 5:** Visual acuity percentile distribution table across age for OU. Data is expressed in LogMAR.

Age (Years)	Percentile Rank						
	5	10	15	50	85	90	95
6	0.185	0.143	0.116	0.017	−0.066	−0.083	−0.108
7	0.162	0.121	0.095	−0.003	−0.084	−0.101	−0.125
8	0.140	0.100	0.074	−0.021	−0.101	−0.118	−0.141
9	0.123	0.084	0.059	−0.036	−0.114	−0.131	−0.154
10	0.109	0.070	0.045	−0.048	−0.126	−0.142	−0.165
11	0.089	0.051	0.027	−0.065	−0.141	−0.157	−0.180
12	0.066	0.029	0.005	−0.085	−0.159	−0.175	−0.197

## Data Availability

The data presented in this study are available on request from the corresponding author. The data are not publicly available due to restrictions included in the informed consent provided by participants.

## Abbreviations

The following abbreviations are used in this manuscript:

BCVA	Best-Corrected Visual Acuity
HCVA	Habitual-Correction Visual Acuity
OD	Right Eye
OS	Left Eye
OU	Oculus Uterque
VA	Visual Acuity
